# Antiplatelet therapy in patients with myocardial infarction with non-obstructive coronary arteries: A clinical perspective

**DOI:** 10.3389/fcvm.2022.1081934

**Published:** 2023-01-09

**Authors:** Wenjie Chen, Yufeng Jiang, Tan Chen, Yafeng Zhou

**Affiliations:** ^1^Department of Cardiology, Dushu Lake Hospital Affiliated to Soochow University, Medical Center of Soochow University, Suzhou Dushu Lake Hospital, Suzhou, Jiangsu, China; ^2^Institute for Hypertension of Soochow University, Suzhou, Jiangsu, China

**Keywords:** coronary artery disease non-obstructive, myocardial infarction, antiplatelet therapy, dual antiplatelet therapy, atherosclerotic plaque, microvascular disease

## Abstract

Myocardial infarction with non-obstructive coronary arteries (MINOCA) is a heterogeneous group of diseases with different pathological mechanisms, and it is uncertain whether the classical secondary prevention and treatment strategies for myocardial infarction in obstructive coronary artery disease (MI-CAD) are appropriate for patients with MINOCA. Therefore the choice of antiplatelet agents and the therapeutic effect may vary depending on the etiology and pathophysiological mechanisms of MINOCA. This requires our clinical and scientific researchers to properly design prospective studies to explore the pathophysiology of MINOCA and its corresponding etiology in greater depth, so as to understand the effectiveness and safety of medical therapies for different etiologies of MINOCA. Although the current observational studies do not show an obvious beneficial effect of antiplatelet therapy on MINOCA. We are eager to conduct specific prospective randomized controlled trials of antiplatelet agents to assess the specificity, efficacy and safety of different types of antiplatelet agents in patients with MINOCA of different etiologies.

## 1. Introduction

Myocardial infarction (MI) is a common cardiovascular disease that affects human life and health; therefore, it is particularly important for the prevention and treatment of MI (1). According to the results of coronary angiography, whether the coronary artery stenosis is ≥ 50%, it is divided into MI with obstructive coronary artery disease (MI-CAD) and MI with non-obstructive coronary arteries (MINOCA) (2). Numerous randomized controlled trials have demonstrated the clear efficacy of antiplatelet agents in the treatment of MI-CAD, and this result is supported by clinical guidelines (3). MINOCA accounts for 6–8% of all MI (1, 4). Depending on the underlying pathophysiological mechanism, MINOCA can be classified into type I and type II MIs, type 1 MIs caused by atherosclerotic plaque disruption and type 2 MIs caused by non-atherosclerotic thrombogenic mechanisms (1). There is increasing evidence that the prognosis of MINOCA is of concern (4, 5), the course of MINOCA is not benign, the all-cause mortality rate in MINOCA patients at 1 year is 4.7% (4), and approximately 23.9% of MINOCA patients experienced a major adverse cardiovascular events (MACE) during 4 years of follow-up (6). The proportion of patients with MINOCA and MI-CAD in cardiac arrest, decreased ejection fraction or heart failure is similar (7). Women with MINOCA show a trend of worse functional outcomes than men (7). MINOCA is a heterogeneous group of diseases with different pathological mechanisms. Therefore, it is uncertain whether the classical secondary prevention and treatment strategies for MI-CAD are appropriate for patients with MINOCA (1, 8). Whereas most MINOCA management in clinical practice is based on imitation and analogy with studies in patients with MI-CAD (9), it is unknown whether antiplatelet agents are efficacious in MINOCA patients, and there are no clear randomized controlled trials (RCTs) evaluating the role of antiplatelet therapy in a cohort of patients with MINOCA or specifically analyzing the specific etiology of MINOCA. Therefore, the choice of antiplatelet agents and the efficacy of treatment may vary depending on the etiology and pathophysiological mechanisms of MINOCA.

## 2. MINOCA-related etiology, pathogenesis, and clinical trials

### 2.1. Types of antiplatelet drugs

Antiplatelet drugs can be divided into those that block membrane receptors and those that block intracellular signaling. The former are divided into P2Y12 receptor blockers, GPIIb/IIIa receptor inhibition and protease-activated receptor 1 PAR1 inhibitors; the latter include COX-1 inhibitors, PDE5 inhibitors and PDE3 inhibitors (10–12).

P2Y12 receptor blockers are used to inhibit the activation of the glycoprotein IIb/IIIa receptor complex by binding to the adenosine diphosphate (ADP) P2Y12 receptor on the platelet surface, as it plays an important role in platelet aggregation and thrombosis. Representative drugs for P2Y12 receptor blockers include oral clopidogrel and prasugrel, parenteral use of Cangrelor and Selatogre (10); The GPIIb/IIIa receptor is one of the key integrins involved in platelet aggregation and therefore also involved in thrombosis, represented by drugs including Abciximab, Eptfibatide, and Tirfiban (11), protease-activated receptor 1 PAR1 inhibitors (e.g., vorapaxar), which by antagonizing PAR-1 receptor expression can inhibit the platelet activation process and thus exert antithrombotic efficacy (12).

COX-1 inhibitors modulate the synthesis of platelet cyclooxygenase and reduce the level of prostacyclin (PGI) as well as TXA2 in the blood, thus improving the platelet agglutination state (11), for example, Aspirin is a commonly used COX-1 inhibitor; common drugs for PDE5 inhibitors include Dipyridamole, Cilostazol is a commonly used PDE3 inhibitor (11).

Clinical experts generally believe that the use of platelet drugs should follow the individualized medication. According to the etiology of MINOCA patients, appropriate antiplatelet drugs should be selected, and appropriate drug dosage and treatment duration should be adjusted, which is important to effectively improve the prevention and treatment of thrombotic diseases. Dual antiplatelet therapy (DAPT), aspirin plus an oral P2Y12 adenosine diphosphate (ADP) receptor inhibitor, is the standard of care after PCI that is widely recommended to reduce cardiovascular events, including in-stent thrombosis, MI, and cardiogenic death (3).

### 2.2. Etiology of MINOCA

The etiology of MINOCA can be divided into coronary atherosclerotic and coronary non-atherosclerotic (8, 13). Coronary atherosclerotic plaque rupture has been typical of the pathophysiology of MI, and plaque rupture may induce thrombosis, which can lead to infarction by distal embolization or inducing coronary spasm (13). Plaque rupture is a common cause of atherosclerotic thrombotic events (14) and may also be a common cause of MINOCA (13). In addition to plaque rupture, plaque erosion is the second most common cause of atherosclerotic thrombosis, which is involved in larger vessel lumens and more platelet-rich thrombi (15),and may fit the definition of type 1MI (1).

If coronary embolism or *in situ* thrombosis leads to a non-obstructive angiographic pattern, it may be present in the form of MINOCA (16). The common mechanism of coronary thrombosis-associated hypercoagulable disease associated with MINOCA is microvascular obstruction due to microthrombi. The key receptors and pathways for microthrombosis require platelet involvement. Microembolism leads to platelet and inflammatory cell activation and vasospasm, which reduces coronary blood flow with mechanical occlusion of the microcirculation (17), and exhibits increased platelet activation in the acute phase or even at 1-month follow-up. Epicardial coronary spasm (CS) is also a mechanism of MINOCA episodes, and it has been shown that coronary spasm associated with acute myocardial ischemia leads to platelet activation and aggregation in the coronary circulation (18) and may exacerbate symptoms and outcomes. In most cases, Spontaneous Coronary Artery Dissection (SCAD) leads to concomitant significant stenosis (> 50% stenosis) and is therefore an uncommon mechanism of MINOCA (13). The above described ruptures without significant small atherosclerotic plaques, SCAD, pericardial vasospasm and *in situ* thrombosis are all processes with epicardial coronary vascular involvement (13). Therefore, effective intervention measures for epicardial vessel thrombosis may differ from those for microvascular thrombosis.

**FIGURE 1:**
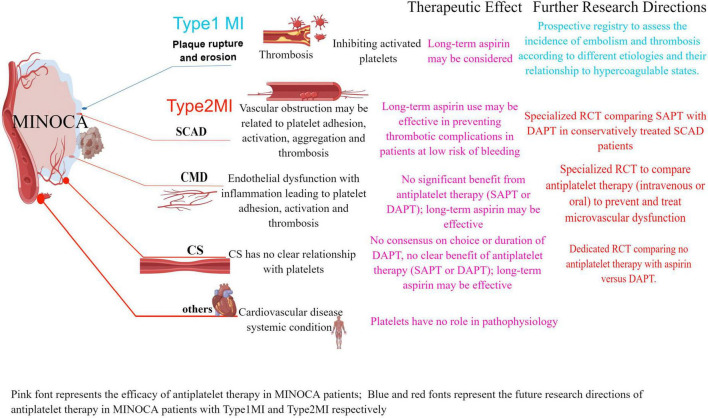
The role of antiplatelet agents in the main etiology of MINOCA according to the 4th UDMI classification, further research directions: dedicated RCTs to evaluate the use of antiplatelet therapy (type, dose, and duration) and its association with the etiology of MINOCA in prospective clinical studies. MINOCA, myocardial infarction with non-obstructive coronary arteries; UDMI, universal myocardial infarction definition; MI, myocardial infarction; SCAD, spontaneous coronary artery dissection; CS, epicardial coronary spasm; CMD, coronary microvascular disease.

Coronary microvascular disease (CMD) is caused by microvascular dysfunction and the initial underlying mechanism of ischemia is endothelial dysfunction (endothelium-dependent dysfunction) (19). The main manifestations are increased microvascular contractility reactivity (possibly mainly related to coronary microcirculation) and decreased microvascular dilation function (20, 21). Coronary microvascular function can be evaluated by coronary angiography-derived index of microvascular resistance (caIMR) in MINOCA patients (20). CMD prevents the necessary increase in coronary flow in response to increased oxygen demand (13) and may lead to myocardial ischemia and vascular inflammation, microembolism with persistent platelet activation leads to an intense inflammatory state (22). As per 4th Universal Definition of Myocardial Infarction, the Tako-tsubo syndrome is not considered a form of MI by the new definition (1). The distinction between MI and TTS could be challenging (1). There are still unknown mechanisms related to heart and physical conditions behind some cases of MINOCA (2) (Figure 1 by Figdraw).

### 2.3. Clinical guidelines and expert recommendations on the etiology associated with MINOCA

In 2017, the European Society of Cardiology (ESC) Working Group on Cardiovascular Pharmacotherapy published a consensus document defining MINOCA, describing the clinical features and mechanisms of MINOCA and facilitating research into its potential mechanisms and treatment (17). In 2019, the American Heart Association (AHA) published a scientific statement document on MINOCA (16). According to the AHA and ECS clinical guidelines, long-term aspirin is recommended for plaque rupture; Antiplatelet or anticoagulation therapy is recommended for coronary embolism or *in situ* thrombosis (16); Antiplatelet agents are not recommended for CS (16); There are AHA and ESC-related clinical guidelines that do not specify the exact efficacy of DAPT and duration for SCAD. Aspirin may have a preventive effect on thromboembolism formation in SCAD (16, 23); There are some small studies that suggest routine antianginal with dipyridamole may be of value in the treatment of CMD (16).

### 2.4. Studies related to antiplatelet agents for MINOCA

In an observational study of a large cohort of patients from the SWEDEHEART registry assessing drug therapy and outcomes in MINOCA, Although the exact duration of DAPT in patients was uncertain in the study by Lindahl et al., the absence of underlying etiology findings limits the effects of DAPT in the study population; the study concluded that DAPT was not associated with the risk of MACE and bleeding events (6); A single-center retrospective registry of patients with MINOCA treated with DAPT did not reduce MACE (24); A clinical database from the prospective studies of MINOCA patients treated with DAPT had no impact on the reduction of MACE (25); And in the CURRENT-OASIS 7 trial, the intensive dosing strategy did not appear to provide additional benefit or even a deleterious signal compared with the standard clopidogrel-based DAPT regimen (26); DAPT with aspirin and ticagrelor was linked to a significant decrease in thrombus volume and a low rate of events at 1-month follow-up, MACE did not occur in 92.5% of patients with MI caused by plaque erosion who were treated with DAPT without stenting (27). Therefore, DAPT may have a similar effect if individualized treatment is considered for MINOCA patients with underlying etiology of plaque rupture or erosion; Multicenter Italian retrospective registry of patients with MINOCA treated with DAPT did not reduce the incidence of MACE (28). DAPT treatment for patients diagnosed with MINOCA in the Veterans Affairs System was not associated with a reduction in MACE (29). Overall, the relatively small number of patients with MINOCA makes the quality of the findings low, which needs to be confirmed in larger prospective samples (Table 1).

**TABLE 1:** Summary of the relevant studies related to antiplatelet agents for MINOCA.

References	Methodology	Sample size	Follow-up period	Results
Lindahl et al. (6)	Observational study of the SWEDEHEART registry between July 2003 and June 2013 and followed until December 2013	A total of 9,466 consecutive patients with MINOCA, 66.4% were treated with DAPT	4.1 years	DAPT have a null effect [HR 0.90, 95% CI (0.74–1.08)] on 1-year MACE-DAPT was not associated with an increase in bleeding events [HR 1.33, 95% CI (0.73–2.42)]
Abdu et al. (24)	Single-center retrospective registry of patients diagnosed with MINOCA between 2014 to 2018	A total of 259 patients (9.1%) were classified as MINOCA, and 43.1% were treated with DAPT.	2 years	Treatment with DAPT was not associated with a reduction of MACE [HR 1.53, 95% CI (0.78–3.01)].
Paolisso et al. (25)	Patients with MI undergoing early coronary angiography between 2016 and 2018 were extracted from a clinical database of the Bologna University Hospital.	Out of 1,141 MI who underwent coronary angiography, 134 were initially diagnosed as MINOCA, and 42.1% were treated with DAPT.	19.35 ± 10.65 months	Treatment with DAPT was not associated with a reduction in all-cause mortality [HR 0.48, 95% CI (0.14–1.64)] or MACE [HR 0.42, 95% CI (0.14–1.24)]
Bossard et al. (26)	*Post hoc* analysis of the OASIS 7 trial	A total of 23,783 patients with MI and 1,599 (6.7%) with MINOCA were included.	1 years	Intensive dosing strategy did not appear to provide additional benefit or even a deleterious signal
Ciliberti et al. (28)	Multicenter Italian retrospective registry of patients discharged with MINOCA diagnosis from 2012–2018.	A total of 621 patients were included, and 58.8% were treated with DAPT.	90 months	DAPT was not associated with a reduction in all-cause mortality [HR 1.04, 95% CI (0.68–1.59)]
Kovach et al. (29)	Patients who underwent coronary angiography in the Veteran Affairs system were diagnosed as MINOCA	A total of 1,986 patients were diagnosed as MINOCA, and 20% were treated with DAPT.	1 years	Treatment with DAPT was not associated with a reduction in MACE [HR 1.02, 95% CI (0.58–1.80)].

## 3. Discussion

In the last decade, there have been developments in the understanding of risk factors, diagnosis, morbidity and prognosis, and underlying pathophysiological mechanisms of MINOCA, but fundamental gaps remain in our understanding of this heterogeneous entity (8). Although there have been several studies on the therapeutic efficacy of antiplatelet agents in patients with MINOCA, there is still a lack of convincing evidence to demonstrate the beneficial effect of DAPT in the presence of small plaque rupture in non-significant stenosis and non-thrombotic coverage.

First, the pathophysiological mechanisms of platelet involvement in certain etiologies (e.g., coronary microcirculatory dysfunction) are not fully understood, nor is there a clear relationship between certain etiologies (e.g., CS, SCAD) and platelet function, thus considering the mismatch between different types of antiplatelet agents and the cause of MINOCA, and there are no relevant studies addressing the different causes of MINOCA. This may also affect the variability of the results. Considering that the etiology of MINOCA is not fully elucidated, the selection of the optimal type of antiplatelet regimen is still difficult, which requires further exploration by clinical and research workers to understand the relationship between platelets and MINOCA and to identify potential therapeutic targets.

Second, the use of antiplatelet agents is not strictly regulated, and current studies on antiplatelet agents for the treatment of MINOCA have used oral antiplatelet agents, but there is no preliminary trial to evaluate the safety and efficacy of parenteral antiplatelet drugs in antiplatelet therapy. Moreover, the dose and duration of antiplatelet use and the combination of drugs have not been uniformly specified, which does not completely indicate that antiplatelet drugs are completely ineffective in MINOCA and require further and more refined research exploration.

Again, although there is no definitive clinical evidence that antiplatelet drugs have significant efficacy for MACE in MINOCA, there are no clear indications that antiplatelet drugs will bring irreversible and serious clinical consequences to patients with MINOCA. Therefore, the use of antiplatelet agents is not currently prohibited for the clinical treatment of MINOCA, but is based on the recommendations of registries and experts. Since the current research evidence on the role of antiplatelets in patients with MINOCA comes from registries or secondary analyses of RCTs, the current low-quality evidence shows that it is unclear whether DAPT is an effective target in the treatment of MINOCA patients, especially in non-atherosclerotic causes. But at present, it cannot be considered that DAPT has nothing to do with the better outcome of MINOCA patients. There is an urgent need for investigators to conduct specific prospective randomized controlled trials on antiplatelet agents in patients with MINOCA of different etiologies, in order to assess the specificity, efficacy and safety of different types of antiplatelet agents in patients with MINOCA of different etiologies.

In addition, due to the different treatment duration of antiplatelet drugs for MINOCA patients, the treatment effect may also be different, and the incidence of MACE may also be different, which requires further clinical research by our researchers. Although some studies have classified MINOCA into ST-segment elevation-MINOCA (STE-MINOCA) and non-ST-segment elevation-MINOCA (NSTE-MINOCA) based on whether ST segment of ECG is elevated or not, and explored the differences between patients with STE-MINOCA and NSTE-MINOCA who exhibit similar clinical features treated with DAPT. Compared with STE-MINOCA, NSTE-MINOCA seemed to be associated with worse long-term clinical outcomes (30), but the clinical sample size is small and a larger sample size is needed to further confirmation.

The current information available regarding the role of antiplatelet agents in patients with MINOCA remains uncertain; neither is it clear whether it is an effective target, nor is it significantly associated with adverse consequences. Therefore, in the current clinical work, the treatment of MINOCA with antiplatelet agents is based on the recommendations of registries and experts, and the prognosis may be clinically significant for patients with MINOCA. The studies currently conducted are usually in small and selected study populations, and there is a lack of sufficiently large and appropriate study populations and prospective randomized controlled trials, which requires our clinical and research staff to properly design prospective studies to explore the pathophysiology of MINOCA and the corresponding etiology in greater depth, in order to understand the effectiveness and safety of medical therapies for different etiologies of MINOCA.

## 4. Future perspective

In the past few years, researchers have been committed to the diagnosis and understanding of MINOCA. However, the trend in the next few years is that a lot of research will be devoted to the exploration of the pathogenesis of MONOCA and the safety and effectiveness of potential treatment schemes. It is necessary to improve the prognosis and survival rate of MINOCA patients. It is known that NCT04538924 and NCT04850417 are ongoing clinical trials to evaluate the role of antiplatelet therapy in MINOCA. It is important to determine the underlying cause, since prognosis and treatment vary depending on the underlying cause. The use of statins and angiotensin-converting enzyme inhibitor/angiotensin II receptor antagonist has been shown to reduce mortality in MINOCA patients. While aspirin, clopidogrel and beta-blocker have not shown any significant mortality benefit. Since there is no detailed evidence to demonstrate the beneficial clinical outcome of antiplatelet therapy in patients with MINOCA, based on the recommendations of registries and experts, we could still perform antiplatelet therapy in patients with MINOCA in our clinical work. Therefore, further randomized clinical trials are necessary to evaluate the treatment of MINOCA patients.

## Author contributions

WC wrote the first draft of the report. YJ, TC, and YZ helped to wrote the final version. All authors read and met the criteria for authorship and agreed with the results and conclusion of the report.
